# Novel Cerium‐Based p‐Dopants with Low Parasitic Absorption for Improved Organic Devices

**DOI:** 10.1002/advs.202414959

**Published:** 2025-02-18

**Authors:** Stephanie A. Buchholtz, L. Conrad Winkler, Maximilian F. X. Dorfner, Fred Kretschmer, Anncharlott Kusber, Léonard Y. M. Eymann, Theresa Schmidt, Hans Kleemann, Johannes Benduhn, Frank Ortmann, Karl Leo

**Affiliations:** ^1^ Dresden Integrated Center for Applied Physics and Photonic Materials (IAPP) and Institute of Applied Physics Technische Universität Dresden Nöthnitzer Straße 61 01187 Dresden Germany; ^2^ Department of Chemistry TUM School of Natural Sciences Technische Universität München Lichtenbergstr. 4 85748 Garching Germany; ^3^ CREDOXYS GmbH Institute for Applied Physics Technische Universität Dresden Nöthnitzer Straße 61 01187 Dresden Germany; ^4^ Micro‐ and Nanoelectronic Systems (MNES) TU Ilmenau Institute for Micro‐ and Nanoelectronics Technische Universität Ilmenau Gustav‐Kirchhoff‐Str.1 98693 Ilmenau Germany

**Keywords:** doping, hole transport layers, organic photodetectors, organic semiconductors, UV–vis–NIR absorption

## Abstract

High electrical conductivity and improved charge carrier injection enabled by molecular doping are pivotal for high‐performance, energy‐efficient, and stable organic optoelectronic devices. Molecular doping is a key element in device design and manufacturing of active‐matrix organic light‐emitting diode displays, a multi‐billion dollar market. However, it is an inherent feature of state‐of‐the‐art small molecule dopants and their charge‐transfer complexes to strongly absorb in the visible and near‐infrared spectral range. This parasitic effect results in absorption losses, reducing the performance in light‐harvesting and light‐emitting applications. Here, a novel class of vacuum‐processable cerium‐based p‐dopants with excellent processing properties and competitive doping strength even in organic hole transport layers with low‐lying valence levels is presented. A substantial reduction in parasitic absorption for layers doped by the new dopants in the visible and near‐infrared range is found. The reduced polaron absorption of the dopant anions is in excellent agreement with theoretical simulations. By incorporating these dopants into near‐infrared narrowband organic photodetectors, the specific detectivity can be increased by one order of magnitude compared to devices with the established dopant 1,3,4,5,7,8‐hexafluorotetracyanonaphthoquinodimethane (F_6_‐TCNNQ). The decreased parasitic absorption yields optical‐microcavity‐enhanced photodetectors with significantly reduced full‐width at half maximum, paving the way toward more efficient and wavelength‐selective infrared detectors.

## Graphical abstract

This work introduces a novel class of cerium‐based p‐dopants which can significantly reduce the parasitic absorption of organic hole transport layers in the visible and near‐infrared range. The authors show that incorporating these new dopants instead of 1,3,4,5,7,8‐hexafluorotetracyanonaphthoquinodimethane (F_6_‐TCNNQ) in near‐infrared narrowband organic photodetectors yields a higher quality optical‐microcavity, which leads to a one order of magnitude higher specific detectivity.

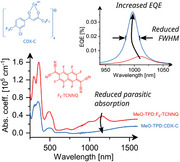

## 1 Introduction

Using electrical doped transport layers in a p‐i‐n diode structure plays a key role for efficient and stable organic optoelectronic devices.^[^ ^1^, ^2^, ^3^, ^4^, ^5^ ^]^ Doping does not only enhance the sheet conductivity and therefore reduce Ohmic losses, but also decreases the barrier width at the contacts and enables a more efficient charge carrier injection.^[^ ^6^, ^7^ ^]^ Furthermore, junctions based on highly p‐ and n‐type doped transport layers enable the formation of efficient generation/recombination contacts in multi‐junction optoelectronic devices such as tandem solar cells. However, doping also induces an additional parasitic absorption due to the generation of broad polaron absorption features in the red and near‐infrared (NIR) spectral range.^[^ ^8^ ^]^ This parasitic absorption can cause significant losses in optoelectronic devices like OLEDs and organic solar cells. It is particularly relevant for NIR organic photodetectors (OPDs), which recently received broad attention due to their competitive performance figures.^[^ ^1^ ^]^ In general, every absorption of the transport layers in the operation wavelength range reduces the efficiency of these devices and, in particular, complicates narrow‐band sensing utilizing cavity‐based OPDs.

**Figure 1:**
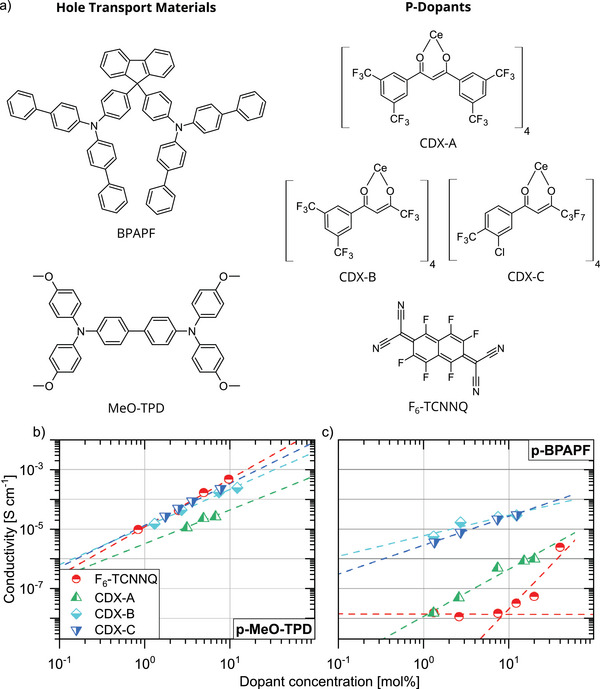
a) Chemical structures of the used hole transport materials (BPAPF and MeO‐TPD), the three tested Ce(IV)‐complexes (CDX‐A, CDX‐B, and CDX‐C), and the reference p‐dopant (F_6_‐TCNNQ). b,c) Electrical conductivity versus dopant concentration for MeO‐TPD and BPAPF doped by the Ce‐based dopants and F_6_‐TCNNQ. The dashed lines are guide to the eye.

Hole transport materials (HTMs) used in common OLED stacks such as N,N,N′,N′‐Tetrakis(4‐methoxyphenyl)‐benzidine (MeO‐TPD) and 9,9‐bis[4‐(N,N‐bis‐biphenyl‐4‐yl‐amino)phenyl]‐9H‐fluorene (BPAPF) (see chemical structures in **Figure** **1**) absorb in the UV range.^[^ ^8^, ^9^ ^]^ However, p‐dopants reported in the literature and the radical cations/anions of hosts/dopants show absorption peaks in both the visible and NIR range. For example, the state‐of‐the‐art p‐dopant NDP9, which shows a broad absorption feature in the visible range, as measured via transmission measurements by Bormann et al.^[^ ^10^ ^]^ Another example is 1,3,4,5,7,8‐hexafluorotetracyanonaphthoquinodimethane (F_6_‐TCNNQ, shown in Figure 1), a well‐known and widely used p‐dopant, which has a dominant absorption peak at around 500 nm, while its radical anions absorb additionally between 800 and 1200 nm.^[^ ^8^ ^]^ An appropriate choice of host and dopant materials for the transport layers and knowledge about their electrical and optical properties is therefore crucial for the development of efficient optoelectronic devices.

In this work, we present a new class of Ce(IV)‐complexes as p‐dopants.^[^ ^11^, ^12^ ^]^ We investigate the electrical and optical properties of three of the novel p‐dopants with conductivity, UV‐vis‐NIR absorption, and temperature‐dependent impedance measurements and find that CDX‐B and CDX‐C perform much better than the well‐established p‐dopant F_6_‐TCNNQ. In particular, they show a substantial reduction in parasitic absorption for layers doped by the new dopants in the visible and near‐infrared range. To prove the excellent performance of the new dopant CDX‐C, cavity‐enhanced narrowband organic photodetectors are realized and compared with the reference dopant F_6_‐TCNNQ. With the reduced parasitic absorption of the new dopant and the excellent electric performance, we achieve a specific detectivity of 7.50 × 10^11^ Jones at 997 nm, which is about one order of magnitude higher than for the reference. Moreover, the improved devices show an excellent full‐width at half maximum of only 24 nm, showcasing the superior absorption properties of these new dopants.

## 2 Results

### 2.1 Introduction to the Materials

The LUMO levels of the prototypes CDX‐A and CDX‐C are obtained from cyclic voltammetry (CV) with 0.00 V versus Fc/Fc^+^ and 0.41 V versus Fc/Fc^+^, respectively, which is about −4.80 and −5.21 eV, assuming a redox potential of ferrocene of −4.8 eV.^[^ ^13^ ^]^ For the commercially available dopant CDX‐B, a LUMO level of +0.35 V versus Fc/Fc^+^ (−5.15 eV) is determined. These numbers indicate CDX‐C and CDX‐B as stronger dopants than F_6_‐TCNNQ (LUMO at 0.24 V vs Fc/Fc^+^), while CDX‐A may be weaker.^[^ ^14^ ^]^ The strong p‐doping capability stems from the high valence states of the metal (i.e., Ce(IV)) as similarly exemplified by dopants based on Re(VI) or Re(VII) oxides.^[^ ^15^, ^16^ ^]^ This property is already known for cerium(IV) ammonium nitrate, which makes it a commonly used oxidant in organic chemistry.^[^ ^17^ ^]^ Taking advantage of this property of Ce(IV) as a strong oxidant serves to create excellent electron acceptors, while the overall doping strength as well as other properties like the processibility in vacuum and solution can be further tuned by variation of the ligands.

As host materials, we use MeO‐TPD and BPAPF. MeO‐TPD has a low ionization energy (IE) of 5.1 eV, which makes it easy to p‐dope this material even with weaker dopants like CDX‐A.^[^ ^6^ ^]^ BPAPF, on the other hand, has a high ionization energy of 5.6 eV.^[^ ^18^ ^]^ Due to its high glass transition temperature of 167 °C and low‐lying HOMO level, it is of particular interest for blue OLEDs.^[^ ^19^, ^20^ ^]^ In addition, we measure the energies of the HOMO levels of the hole transport materials with cyclic voltammetry (CV) for a better comparison with the determined LUMO levels of the dopants, see Figure S1 (Supporting Information). It should be considered that the energy levels determined by CV were measured in solution. That makes a comparison between the materials possible, but it can be expected that the actual energy levels in the thin film deviate from the values determined with CV.

Considering the LUMO levels of the dopants, none of them might be an ideal p‐dopant for BPAPF. However, Nell et al. showed that charge transfer could still be possible even if the acceptor level is higher than the HOMO level of the host due to the disorder in amorphous materials.^[^ ^21^ ^]^ Therefore, the stronger dopants might still be able to dope BPAPF successfully. The chemical structures of all materials and their energy position of the frontier orbitals are shown in Figure 1, **Tables** **1** and **2**.

**Table 1:** Energies of the lowest unoccupied molecular orbitals and molar masses of F_6_‐TCNNQ and the Ce‐based dopants, measured by cyclic voltammetry.

p‐dopant	*E*_LUMO_ [eV]	Molar mass [g mol^−1^]
F_6_‐TCNNQ	−5.04^[^ ^12^ ^]^	362.2
CDX‐A	−4.80	2121.1
CDX‐B	−5.15	1544.7
CDX‐C	−5.21	1810.5

**Table 2:** Ionization energies (IE), measured with ultraviolet photoelectron spectroscopy, and molar masses of the used hole transport materials.

HTM	*IE* [eV]	*E*_HOMO_ [eV]	Molar mass [g mol^−1^]
MeO‐TPD	5.1^[^ ^6^ ^]^	−4.96	608.7
BPAPF	5.6^[^ ^13^ ^]^	−5.29	957.2

The energies of the highest occupied molecular orbitals E_HOMO_ are measured by cyclic voltammetry, see Figure S1 (Supporting Information).

High‐resolution mass spectroscopy data for the new dopants can be found in Figure S2 (Supporting Information). Details about the measurements, device configurations, and setups are summarized in the Experimental Section.

### 2.2 Electrical Conductivity and Activation Behavior

As shown in Figure 1b, MeO‐TPD layers doped with CDX‐C, CDX‐B, and F_6_‐TCNNQ exhibit a comparable specific electrical conductivity at room temperature. The lowest conductivity of doped MeO‐TPD is measured with CDX‐A, presumably the weakest dopant due to its relatively low electron affinity. Nevertheless, given that the HOMO of MeO‐TPD is positioned at −5.1 eV, a continuous increase of conductivity with increasing doping ratio is observed for all dopants tested in this study.^[^ ^6^ ^]^

BPAPF though, has a low‐lying HOMO level, which renders a charge transfer process from the host to the dopant more difficult. Here, BPAPF:CDX‐B and BPAPF:CDX‐C show three‐orders of magnitude higher conductivity than the layers doped by F_6_‐TCNNQ, where the lower limit of detection of the setup is already reached for small doping concentrations, as depicted in Figure 1c. Hereby, the highest reached conductivities at about 12 mol% are 2.9 × 10^−5^, 3.0 × 10^−5^, and 3.3 × 10^−8^ S cm^−1^ for CDX‐B, CDX‐C, and F_6_‐TCNNQ, respectively. This good result of CDX‐B and CDX‐C is even comparable to conductivities achieved with NDP9 in BPAPF.^[^ ^22^ ^]^ They seem therefore indeed to be stronger acceptors than F_6_‐TCNNQ. Surprisingly, even CDX‐A, which has the highest LUMO level measured with CV, achieves an order of magnitude better conductivities than F_6_‐TCNNQ in BPAPF at around 10 mol%. Upon charge transfer from the semiconductor to the Ce‐complex, the transferred electron localizes on the Ce‐center, which is shielded by the surrounding ligands. This shielding effectively prevents significant interaction between the transferred electron and the positive charge generated in the semiconductor, enabling a better charge separation and high conductivity. In addition, the energy levels are measured with CV in solution. The positions of the LUMO and HOMO levels within the layer, influenced by disorder and other factors related to the solid state, are in fact unknown. In particular, the size of the Ce(IV)‐dopants could have a different influence on the energetic landscape of a layer compared to the small and planar F_6_‐TCNNQ. Noticeably, the conductivity increases—depending on the material—either super‐linearly, sub‐linearly, or exponentially with doping concentration. These different activation behaviors can be associated with the location of the energy levels. Therefore, we also investigate the activation energies of the doping process in MeO‐TPD and BPAPF with a temperature‐dependent Mott–Schottky‐analysis for CDX‐C and F_6_‐TCNNQ, which also delivers information about the doping efficiency.^[^ ^23^ ^]^

**Figure 2:**
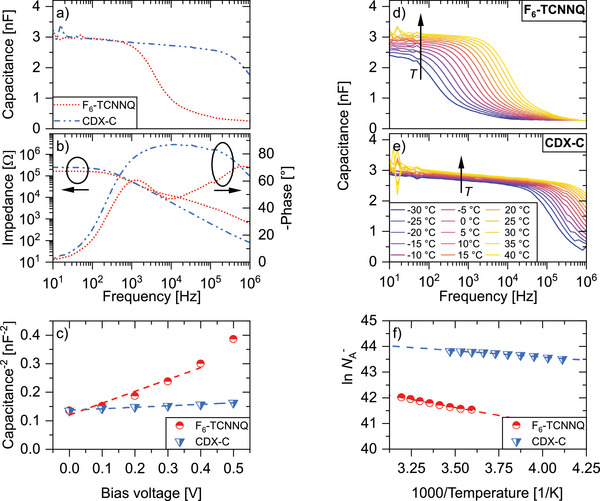
a) Capacitance over frequency and b) Bode plots at 0 V bias voltage and room temperature, c) Mott–Schottky plots at room temperature and a frequency of 1000 Hz (F_6_‐TCNNQ) and 10000 Hz (CDX‐C), d,e) capacitance over frequency for various temperatures at 0 V bias voltage, and f) logarithm of the density of ionized dopants $N_{A}^{-}$ over the inverse of temperature for BPAPF doped by 15 mol% of F_6_‐TCNNQ and CDX‐C.

**Figure** **2**a,b shows the capacitance over frequency and the Bode plots for layers of BPAPF doped by both dopants, where the examined layer is evaporated between two vertical contacts, one of gold and the other of aluminum. A depletion zone is formed at both contacts, but a higher capacitance at the gold contact can be expected due to the larger work function of gold (5.31 eV) compared to aluminum (4.26 eV), which makes it possible to neglect it.^[^ ^24^ ^]^ The capacitance values of the depletion zone established at the aluminum contact can be seen at low frequencies in Figure 2a. At high frequencies, the capacitance drops down due to non‐negligible series resistances. The low‐frequency capacitance is related to the width of the depletion zone and, therefore, the density of ionized dopants. The latter can be determined by performing a Mott–Schottky analysis at a specific constant frequency in the reverse direction, as depicted in Figure 2c. This frequency must be chosen in a way that the phase is about −90° and lies within a plateau of capacitance. Note that the bias voltage range is limited, caused by ohmic leakage currents at higher reverse voltages. Theoretically, we could calculate the doping efficiency now directly by estimating the density of ionized dopants from the Mott‐Schottky analysis and the density of all deposited dopants by knowing the geometry, density of the layer, and the doping concentration. However, the capacitance is not perfectly frequency‐independent as required for the Mott–Schottky analysis even at very low frequencies. In addition, the density of the layer can only be established approximately. To circumvent these problems and uncertainties, we exploit the thermal activation of the doping process. Figure 2d,e shows the temperature‐dependent capacitance over frequency for BPAPF doped by 15 mol% of CDX‐C and F_6_‐TCNNQ. In this way, only the permittivity has to be determined. We estimate *ε*_r_ = 3.1 and *ε*_r_ = 3.2 for MeO‐TPD and BPAPF, respectively (see Figure S3, Supporting Information).

The temperature dependency of the capacitance delivers information about the activation energy *E*_A_. By using a Boltzmann approximation

(1) $$N_{A}^{-}=N_{A}e^{-\frac{E_{A}}{k_{B}T}}$$

and the slopes, obtained from Figure 2f, we determine *E*_A_. The used frequency for the Mott–Schottky analysis at all evaluated temperatures must be the same. Therefore, the suitable temperature range for the fit is limited caused by the temperature dependency of the phase and the frequency selection requirements, see above.

Subsequently, we calculate the doping efficiency:

(2) $$\eta =\frac{N_{A}^{-}}{N_{A}}$$

with the density of ionized dopants *N*_A_^−^ and the density of the deposited dopants *N*_A_. The results for MeO‐TPD and BPAPF are summarized in **Table** **3**, and they quantitatively express the trend, which was already observed in the conductivity curves (see also Figure S3, Supporting Information). CDX‐A is the weakest acceptor and exhibits the lowest doping efficiency in MeO‐TPD. CDX‐C and F_6_‐TCNNQ, on the other hand, are comparably efficient for this host material, since the related activation energies are sufficiently low. However, regarding BPAPF, CDX‐C is a more suitable p‐dopant than F_6_‐TCNNQ, with a doping efficiency of about 19% in comparison to 1%. Considering the significantly lower doping efficiency of F_6_‐TCNNQ in BPAPF, it can be concluded that higher doping concentrations are required to fill the initially appearing charge carrier traps postulated by Tietze et al.^[^ ^25^ ^]^ Accordingly, the dopant performs still in the doping saturation regime even at high concentrations where the Ce‐based dopants reached already the doping reserve regime. This behavior explains the super‐linearly trend for F_6_‐TCNNQ indicated in Figure 1c by dashed lines.

**Table 3:** Activation energies E_A_ and doping efficiencies η, determined by the temperature‐dependent Mott–Schottky analysis.

Sample	E_A_ [meV]	η^[^ ^1^ ^]^
MeO‐TPD:CDX‐A (1.7 mol%)	45	0.17
MeO‐TPD:CDX‐C (1.7 mol%)	9	0.69
MeO‐TPD:F_6_‐TCNNQ (1.7 mol%)	5	0.81
BPAPF:CDX‐C (15 mol%)	42	0.19
BPAPF:F_6_‐TCNNQ (15 mol%)	108	0.01

### 2.3. UV–Vis–NIR Absorption

**Figure 3:**
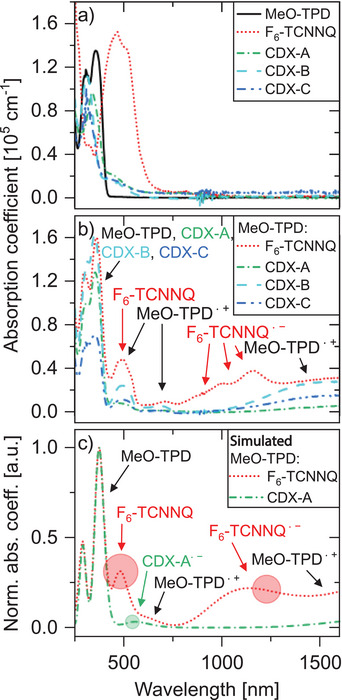
Absorption coefficient of a) neat layers of MeO‐TPD and the p‐dopants F_6_‐TCNNQ, CDX‐A, CDX‐B, and CDX‐C, and b) doped MeO‐TPD (20 mol% for all p‐dopants). c) Simulated normalized absorption for the MeO‐TPD:F_6_‐TCNNQ and MeO‐TPD:CDX‐A system. The circles on the graphs denote the position of computed excited state energies of the dopants and relative dipole strengths (size of the markers). Only the principal transitions in the vis–NIR region that dominate the spectra are shown.

For the investigation of the parasitic absorption, we performed UV–vis–NIR measurements. **Figure** **3**a,b shows the resulting spectra of neat films of MeO‐TPD, CDX‐A, CDX‐B, CDX‐C, and F_6_‐TCNNQ and their blends. We detect the dominant absorption peaks of MeO‐TPD at about 300 and 350 nm and an absorption of the related radical cations at 500 nm, 700 nm, and above 1200 nm. For F_6_‐TCNNQ, the absorption of the neat material is at 500 nm, while its radical anions exhibit three distinguished sub‐gap peaks at about 860, 970, and 1140 nm. The measured absorption spectra of MeO‐TPD and F_6_‐TCNNQ are in agreement with those reported by Tietze et al.^[^ ^8^ ^]^ Using MeO‐TPD:F_6_‐TCNNQ as hole transport layer would lead to a significant parasitic absorption in the entire measured range, while this effect is strongly reduced for layers with the new dopants. The layers with CDX‐A, CDX‐B and CDX‐C have a considerably lower absorption between 400 and 1200 nm.

To support the experimentally found reduced parasitic absorption of the cerium‐based p‐dopants compared to MeO‐TPD:F_6_‐TCNNQ, we theoretically study the gas phase molecules MeO‐TDP, F_6_‐TCNNQ, and CDX‐A using density functional theory (DFT) and linear response time‐dependent density functional theory (TDDFT) as implemented in CP2K.^[^ ^26^ ^]^ We perform ground state relaxations employing the PBE density functional, the TZVP‐MOLOPT‐GTH basis sets in combination with GTH pseudopotentials for the neutral and respectively charged molecules.^[^ ^27^, ^28^, ^29^, ^30^ ^]^ For each of the molecule X, at the PBE optimized geometry, we compute the singlet excited states energies E_X,i_ and their transition dipole moments d_X,I,α_ into direction α, using the CAM‐B3LYP functional, as the TDDFT kernel.^[^ ^31^ ^]^ For this long‐range corrected hybrid density functional theory calculation, we make use of the auxiliary density matrix method using the cpFIT3 auxiliary basis sets for all but the cerium atom in the CDX‐A.^[^ ^32^ ^]^ For the cerium atom only, we use the SZV‐MOLOPT‐SR‐GTH as the auxiliary basis set. In addition, we consider a DFT+*U* correction for the *L* = 2 electrons of the Ce atom with an effective interaction of *U*_eff_ = U − *J* = 7 eV. We tested how sensitive the results are with respect to these choices, by varying *U*_eff_ (and similarly for the *L* = 3 shell) and found that the results are rather insensitive to the specific value of the parameters. To correct for the influence of the dielectric environment, not included in the gas phase calculation, and the known overestimation of excitation energies by using CAM‐B3LYP for TDDFT, we apply a rigid shift of all the energies by −0.3 eV to all excitation energies.^[^ ^33^ ^]^ The theoretical absorption spectrum for the two systems, including different species in the film is calculated to

(3) $$\kappa \left(\omega \right)\propto \sum_{X,i,\alpha }c_{X}d_{X,i,\alpha }^{2}exp\left[{\left(\omega -E_{X,i}\right)}^{2}/\left(2\sigma^{2}\right)\right]$$

where *c_X_* denotes the concentration of the molecular species *X* in the film and the phenomenological broadening with *σ* = 0.2 eV.^[^ ^34^ ^]^ For the comparison to Figure 3b, we also know the concentration of the dopant molecules (*c_D_* = 20 mol%). However, its composition *c_D_* =  *c*_*X*,−_ +  *c*_*X*,0_ by anionic (*c*_*X*,−_) and neutral molecules (*c*_*X*,0_) in the system, is a priori not known. It could be determined as done by Gaul et al. with simulations.^[^ ^35^ ^]^ However, from our calculations it became clear that the absorption peak around 400 nm corresponds nearly exclusively to the neutral MeO‐TDP. This allows estimating the relative concentration of neutral versus charged MeO‐TDP molecules from the transition dipole moments and the relative peak heights of the excitations around 400 nm and around 1650 nm in Figure 3b. With *c*_*MeO* − *TPD*, +_ =  *c*_*X*,−_, we estimate *c*_*F*6 − *TCNNQ*, −_ ≈ 10 mol%, whereas *c*_*CDX* − *A*, −_ ≈ 2 mol%. Note that the resulting ratio $\frac{c_{CDX-A,-}}{c_{F6-TCNNQ,-}}=0.2$ consistently reproduces the ratio of the doping efficiencies from Table 3. Using these values, we compute the normalized absorption spectrum of the mixtures and plot it in Figure 3c. Peak labels indicate the charging state and the molecular species for clarity. The resulting spectra are in very good agreement with the experimental result shown in Figure 3b.

From our simulations, we can identify two reasons for the strongly reduced parasitic absorption in the MeO‐TDP:CDX‐A system compared to MeO‐TDP:F_6_‐TCNNQ. The first reason is based on the reduced interaction strength of the neutral and charged MeO‐TDP:CDX‐A compound with visible light. While, the neutral CDX‐A molecule has no bright state in the energetic region below the MeO‐TDP absorption around 400 nm, the neutral F_6_‐TCNNQ molecule hosts a strong dipole transition around 500 nm, giving rise to the bright absorption feature of the MeO‐TDP:F_6_‐TCNNQ system in this wavelength range. Similarly, also the negatively charged F_6_‐TCNNQ molecule contributes another strong transition in the NIR range around 1100 nm. In contrast, the CDX‐A anion has only semi‐bright states (modest oscillator strength) around 550 nm and no further excitations in the considered spectral range. The calculated dipole strengths of the dopants are summarized in **Table** **4**.

**Table 4:** Theoretically calculated dipole strengths of the dopants.

Molecule	E [eV]	λ [nm]	Dipole strength [e a_0_]
CDX‐A^•^ ‾	2.264	547.6	3.317
	2.283	543.1	5.346
	2.285	542.6	0.254
	2.288	541.9	5.811
F_6_‐TCNNQ	2.559	484.5	36.994
F_6_‐TCNNQ^•^ ‾	1.010	1227.6	29.950
	2.659	466.3	2.124

The second reason for the reduced parasitic absorption is the different doping efficiencies of the two systems. This results in smaller concentrations of the already weaker absorbing CDX‐A species, which further reduces the absorption.

From a more general perspective, the theoretical study of the absorption of MeO‐TPD:CDX‐A demonstrates, representatively also for the other Ce‐based dopants, that no further absorption peaks are to be expected in the measured wavelength range, which can be particularly beneficial for applications in the visible and NIR wavelength range.

### 2.3 Application in Narrowband Near‐Infrared Organic Photodetectors

**Figure 4:**
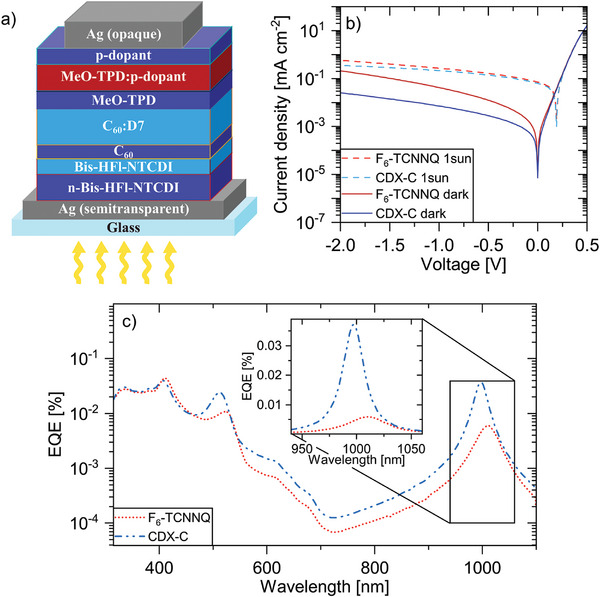
a) Stack structure of the devices with F_6_‐TCNNQ and CDX‐C as p‐dopant (details on the stack are given in the Experimental Section). b) *I*–*V* characteristics under dark conditions and illumination with 1 sun. c) Comparison of the EQE using F_6_‐TCNNQ and CDX‐C, measured at 0 V and enlarged resonance peaks.

As shown above, the new dopants show significantly reduced parasitic absorption and work as efficient electron acceptors. As a final step, we demonstrate the enhancement of an optoelectronic device's efficiency. For this purpose, we investigate organic photodetectors (OPDs) within an optical microcavity to improve the external quantum efficiency (EQE) as shown by similar approaches by Wang et al., Kaiser et al., and Siegmund et al.^[^ ^1^, ^36^, ^37^, ^38^ ^]^ Hereby, we achieve a resonance wavelength at about 1000 nm. The structure of the devices is shown in **Figure** **4**a. For this purpose, we use MeO‐TPD:CDX‐C and MeO‐TPD:F_6_‐TCNNQ, both with a doping concentration of 7 mol%, as hole transport layers. As active layer, we utilize the fullerene C_60_ with the high HOMO donor D7, which was described earlier by Kaiser et al., which leads to charge‐transfer state absorption in the near infrared wavelength range.^[^ ^37^ ^]^ To achieve narrowband signals and to enhance the rather weak charge‐transfer absorption, an optical cavity is used, where the bottom contact is semitransparent for efficient incoupling and the top one is opaque. The EQEs of both devices are depicted over the wavelength in Figure 4c, and the results for the resonance peaks are summarized in **Table** **5**. The full width at half maximum (FWHM) at the resonance wavelength of the CDX‐C sample is 8 nm less than the FWHM of the F_6_‐TCNNQ sample, which can be attributed to a lower parasitic absorption per roundtrip of the photons. This higher quality microcavity results in almost an order of magnitude higher EQE.

**Table 5:** Performance characteristics of the OPDs with F_6_‐TCNNQ and CDX‐C as p‐dopants for MeO‐TPD.

p‐Dopant	Resonance wavelength [nm]	FWHM [nm]	EQE_peak_ [%]	NSD [pA Hz^−1/2^]	D*_real_ [10^11^ Jones]	LDR [dB]	*f*_−3 dB_ [kHz]
F_6_‐TCNNQ	1010	32	0.6	0.14	0.88	160	170
CDX‐C	997	24	3.7	0.11	7.50	160	125

This enhancement of all related OPD characteristics is clearly related to the lower parasitic absorption of the hole transport layer with the Ce‐based dopant. Additionally, we find a lower dark current and slightly lower noise spectral density (NSD) for the device with CDX‐C, as shown in Figures 4b and S4 (Supporting Information). Overall, this leads to a specific detectivity of 7.50 × 10^11^ Jones for the devices with CDX‐C at zero bias (based on noise measurements), which is one order of magnitude better than the reference devices with F_6_‐TCNNQ. Furthermore, both devices show an excellent linear dynamic range (LDR) of 160 dB and reasonable cut‐off frequencies in the range of >100 kHz, see Figure S5 (Supporting Information).

All in all, the result is very satisfactory. Only by changing the p‐dopant of the HTL, it is possible to achieve a higher quality microcavity and thus a significantly improved specific detectivity. Indeed, the improved device shows a good result regarding its FWHM and detectivity, even in comparison to similar state‐of‐the‐art devices.^[^ ^37^, ^38^, ^39^ ^]^ Kaiser et al. for example achieved a FWHM of just 45 nm with a similar device and the Donor D8 at a resonance peak of 1032 nm.^[^ ^37^ ^]^

Based on this result, we expect an improvement in performance also for other optoelectronic devices, such as organic solar cells, which will be able to absorb more photons due to reduced absorption losses in hole transport layers.

## 3 Conclusion

In summary, we present three new Ce(IV)‐complexes which work as efficient p‐dopants, being able to dope hole transport materials with low‐lying HOMO levels like BPAPF. In addition, we find a significantly reduced parasitic absorption in comparison to F_6_‐TCNNQ for all investigated novel dopants in the visible and NIR range, since their radical anions do not show additional absorption features in this spectral range, which was rationalized by the absence of strong optical transitions for the anions in contrast to F_6_‐TCNNQ. Finally, we prove that this reduced parasitic absorption is the key to improve the performance of narrowband near infrared organic photodetectors. Utilizing CDX‐C, we achieve a specific detectivity of 7.50 × 10^11^ Jones (based on noise measurements) at 997 nm, one order of magnitude higher than the reference device with F_6_‐TCNNQ. Moreover, the reduced parasitic absorption in the CDX‐C doped MeO‐TPD layer leads to a narrowband detection down to 24 nm, which is comparable to state‐of‐the‐art devices. We expect that this new dopant class will lead to significantly improved optoelectronic devices due the superior doping properties combined with low parasitic absorption.

## 4 Experimental Section

### Sample Fabrication

Thin layers were produced by thermal vapor phase deposition under high vacuum conditions with a base pressure of about 10^−7^ mbar (Kurt J. Lesker Company GmbH, Germany). The layer thicknesses and deposition rates were controlled and monitored via quartz crystal microbalances (QCMs). Dopant and host materials were co‐evaporated to achieve homogeneous doped layers. To determine the total layer thickness, it was assumed that the density of the layer is approximately the density of the host material. The substrate cleaning procedure included an ultrasonic bath with ethanol, acetone, and isopropanol. As hole transport materials, MeO‐TPD (N,N,N′,N′‐Tetrakis(4‐methoxyphenyl)benzidine, 2× sublimed quality, TCI Deutschland GmbH, Germany) and BPAPF (9,9‐Bis[4‐(N,N‐bis‐biphenyl‐4‐yl‐amino)phenyl]‐9H‐fluorene, 2× sublimed quality, Lumtec, Taiwan) were used. For the active layer of the OPDs, C_60_ (3× sublimed quality, Nano‐C, USA) and the absorber D7 (2,2′,6,6′‐tetrathienyl‐4,4′‐bithiopyranylidene, 1× sublimed quality, IAPP Labs, Germany) were used. The corresponding electron transport layer was produced with Bis‐HFl‐NTCDI (N,N‐Bis(fluoren‐2‐yl)‐naphthalenetetracarboxylic diimide, 2× sublimed quality, Fakultät Angewandte Chemie Technische Hochschule Nürnberg Georg Simon Ohm, Germany) and the n‐dopant W_2_(hpp)_4_ (tetrakis(1,3,4,6,7,8‐hexahydro‐2H‐pyrimido[1,2‐a]pyrimidinato)ditungsten (II), IAPP Labs, Germany). The reference p‐dopant F_6_‐TCNNQ (1,3,4,5,7,8‐hexafluorotetracyanonaphthoquinodimethane, used as received) was delivered by Novaled AG (Germany). The Ce‐based p‐dopants CDX‐A, CDX‐B, and CDX‐C were produced by CREDOXYS GmbH, Dresden, Germany (www.credoxys.com, 1× sublimed quality, purity >99% [HPLC]). The sublimation procedure was done in ultrahigh vacuum at a pressure below 10^−7^ mbar.

All samples except the absorption samples were encapsulated with a glass cover and UV‐hardened epoxy resin (Nagase ChemteX XRN 5592, Japan) in a nitrogen atmosphere directly after the manufacture to minimize the influence of oxygen and moisture.

### Conductivity

The specific electrical conductivity was determined by performing lateral sheet resistance measurements at 50‐nm‐thick organic layers on glass substrates with 50‐nm‐thick gold contacts. Hereby, the channel width was 5 mm and the channel length was varied with 0.1, 0.2, and 0.3 mm. Layers with various doping concentrations between about 1 and 40 mol% were produced. As voltage source, a Keithley 236 source measure unit was taken and using a metal box reduced the electromagnetic noise during the measurements. The measurements were controlled by the software “SweepMe!” and performed in ambient atmosphere.

### Cyclic Voltammetry

The cyclic voltammetry measurements were performed under inert atmosphere of dinitrogen at scan rates between 50 and 1000 mV s^−1^. 0.1 m of [Bu_4_N][PF_6_] (over 98.0% purity, TCI Deutschland GmbH, Germany) was used as the electrolyte in degassed and anhydrous MeCN as solvent (99.9% purity, extra dry over molecular sieve, AcroSeal packed, Thermo Fischer Scientific). The voltammograms were carried out referenced to the external standard ferrocene/ferrocenium (Fc/Fc^+^) couple (99% purity, Alfa Aesar GmbH & Co. KG, Germany). The setup includes a platinum working electrode (surface: 3.142 mm^2^), a platinum counter electrode (surface: 40 mm^2^), and silver wire (diameter: 2 mm) coated with AgCl as the reference electrode. As potentiostatic device, a Metrohm PGSTAT30 (Metrohm Autolab B. V., Netherlands) controlled by the software NOVA 2.1.3 (Metrohm Autolab B. V., Netherlands) was used.

### Impedance

Samples were prepared with vertical contacts with an organic layer thickness of 100 nm for impedance spectroscopy measurements. The bottom and top contacts were made of 30 nm gold on 3 nm chromium and aluminum, respectively. On each substrate, four pixels with different areas were deposited. The measurements were carried out on pixels with an area of 0.9 mm^2^ for the BPAPF and 1.7 mm^2^ for the MeO‐TPD samples. An Autolab potentiostat/galvanostat (PGSTAT302N, Metrohm Autolab B.V., Netherlands) was used to measure the impedance while applying an alternating voltage 𝑣 = 𝑉_DC_ + 𝑉_AC_ sin (𝜔𝑡). Hereby, the amplitude of *V*_AC_ must be smaller than the thermal voltage. Therefore, 20 mV was chosen. By assuming the layer to behave like a parallel connection of a resistor and a capacitor, the capacitance was calculated by 𝐶 = −ℑ(𝑍) / (ω|𝑍|^2^) with the angular frequency ω = 2𝜋*f*. Frequency‐dependent measurements between 10 Hz and 1 MHz and measurements at constant frequencies and a variable bias voltage *V*_DC_ from 0 to 0.5 V in reverse direction were performed. The impedance measurements were controlled by the software Nova 2.1.3 (Metrohm Autolab B.V., Netherlands). For an additional temperature variation, a Peltier cryostat (PK3 228 A 1614 H200, Peltron GmbH, Germany) with a temperature controller from BelektroniG (HAT Control‐K10, BelektroniG GmbH, Germany) was used, which was controlled by the software “SweepMe!” (SweepMe!, Germany).

### Absorption

To investigate the absorption, doped MeO‐TPD layers with a thickness of 120 nm and neat dopant and host layers with 30 nm thickness were evaporated on quartz substrates. High doping concentrations of 20 and 40 mol% were used to examine the influence of the dopants. The spectra were detected with a SolidSpec‐3700 UV–vis–NIR spectrometer from Shimadzu. For this setup, the wavelength accuracy specified by the manufacturer is ±0.2 and ±0.8 nm in the UV–vis and the NIR range, respectively. Direct transmission and integrated reflection spectra were detected separately for each sample using an integration sphere for the latter.

It is noted that the layer thickness was measured with quartz crystal microbalances assuming the density of the host material as density of the overall layer. The high molecular weight of the Ce‐based dopants could lead to an underestimation of the layer thickness. The absorption coefficient may, therefore, differ slightly in its absolute value.

### Organic Photodetectors

Organic narrowband photodetectors were produced by thermal evaporation, as described above, for the near‐infrared range with an optical microcavity and a resonance wavelength of about 1000 nm. These detectors are based on intermolecular charge‐transfer absorption as introduced by Siegmund et al.^[^ ^38^ ^]^ Hereby, D7 was used as donor for the active layer.^[^ ^37^ ^]^ The overlap area of the electrodes and, therefore, the active area was 6.44 mm^2^. MeO‐TPD served as hole transport material and was doped by F_6_‐TCNNQ and CDX‐C, in each case with 7 mol%.

The current–voltage characteristics were taken by a source measurement unit (Keithley SMU 2400) under dark conditions and illumination. For the latter, an intensity of 100 mW cm^−2^ was set with a sun simulator (Solarlight Company Inc., USA) controlled by a Hamamatsu S1337 silicon photodiode.

The EQE was measured using the monochromatic light of a broadband Xe lamp (Oriel Xe Arc‐Lamp Apex Illuminator combined with a Cornerstone 260 1/4 m monochromator (Newport, USA)), chopped at a frequency of 172 Hz and illuminating the devices under normal incidence. The signal of the devices was recorded by a lock‐in amplifier (Signal Recovery SR 7265). The devices were masked to avoid edge effects with an area of 2.78 mm^2^.

In addition, sensitive external quantum efficiency (sEQE) measurements were performed with a chopped monochromatic quartz halogen lamp (140 Hz, 200 W with a double monochromator (Bentham SHD‐300A, QD‐Europe)), which illuminated the devices under normal incidence. The current was detected with a lock‐in amplifier (Stanford Research SR830, USA) and preamplified (Stanford Research SR860, USA).

The transient response of the devices was measured after preamplification (DHPCA‐100, FEMTO Messtechnik GmBH) with a lock‐in amplifier (Stanford Research SR860, USA). The light source was an LED (M660L4, Thorlabs) with an LED driver (BLS‐1000‐2, Mightex Systems Canada) at various frequencies regulated with a lock‐in amplifier (Stanford Research SR860, USA).

To obtain the noise spectral density, a low‐noise current–voltage preamplifier (DHPCA‐200, FEMTO Messtechnik GmbH, Germany) was connected to the devices to measure the noise current. A low‐noise oscilloscope (DPO7354C, Tektronix, USA) with a sampling rate of 10 MS s^−1^ and a high bandwidth recorded the signal. Afterward, the signal was analyzed with the Welch method, transforming the data to the frequency space.^[^ ^40^, ^41^ ^]^

## Conflict of Interest

Léonard Y. M. Eymann and Karl Leo are shareholder of CREDOXYS GmbH, a spin‐off company of TU Dresden developing new dopant materials.

## Supporting information



Supporting Information

## Data Availability

The data that support the findings of this study are available from the corresponding author upon reasonable request.
