# Supplementary material for: Novel Cerium‐Based p‐Dopants with Low Parasitic Absorption for Improved Organic Devices
Source: Adv Sci (Weinh). 2025 Feb 18;12(14):2414959. doi: 10.1002/advs.202414959 (PMC11984852; doi:10.1002/advs.202414959)
Supplement: Supplementary file 1 — Supporting Information [file ADVS-12-2414959-s001.docx]

Supporting Information

Novel Cerium-Based p-Dopants with Low Parasitic Absorption for Improved Organic Devices

Stephanie A. Buchholtz^1*^, L. Conrad Winkler^1^, Maximilian F. X. Dorfner^2^, Fred Kretschmer^1^, Anncharlott Kusber^1^, Léonard Y. M. Eymann^3^, Theresa Schmidt^3^, Hans Kleemann^1, 4^, Johannes Benduhn^1^, Frank Ortmann^2^, and Karl Leo^1*^

^1^Dresden Integrated Center for Applied Physics and Photonic Materials (IAPP) and Institute of Applied Physics, Technische Universität Dresden, Nöthnitzer Straße 61, 01187 Dresden, Germany

^2^TUM School of Natural Sciences, Department of Chemistry, Technische Universität München, 85748 Garching b. München, Germany

^3^CREDOXYS GmbH, Institute for Applied Physics, Technische Universität Dresden, Nöthnitzer Straße 61, 01187 Dresden, Germany

^4^Micro- and Nanoelectronic Systems (MNES), TU Ilmenau, Institute for Micro- and Nanoelectronics, Technische Universität Ilmenau, Gustav-Kirchhoff-Str.1, 98693, Ilmenau, Germany

E-mail: stephanie_anna.buchholtz@tu-dresden.de, karl.leo@tu-dresden.de


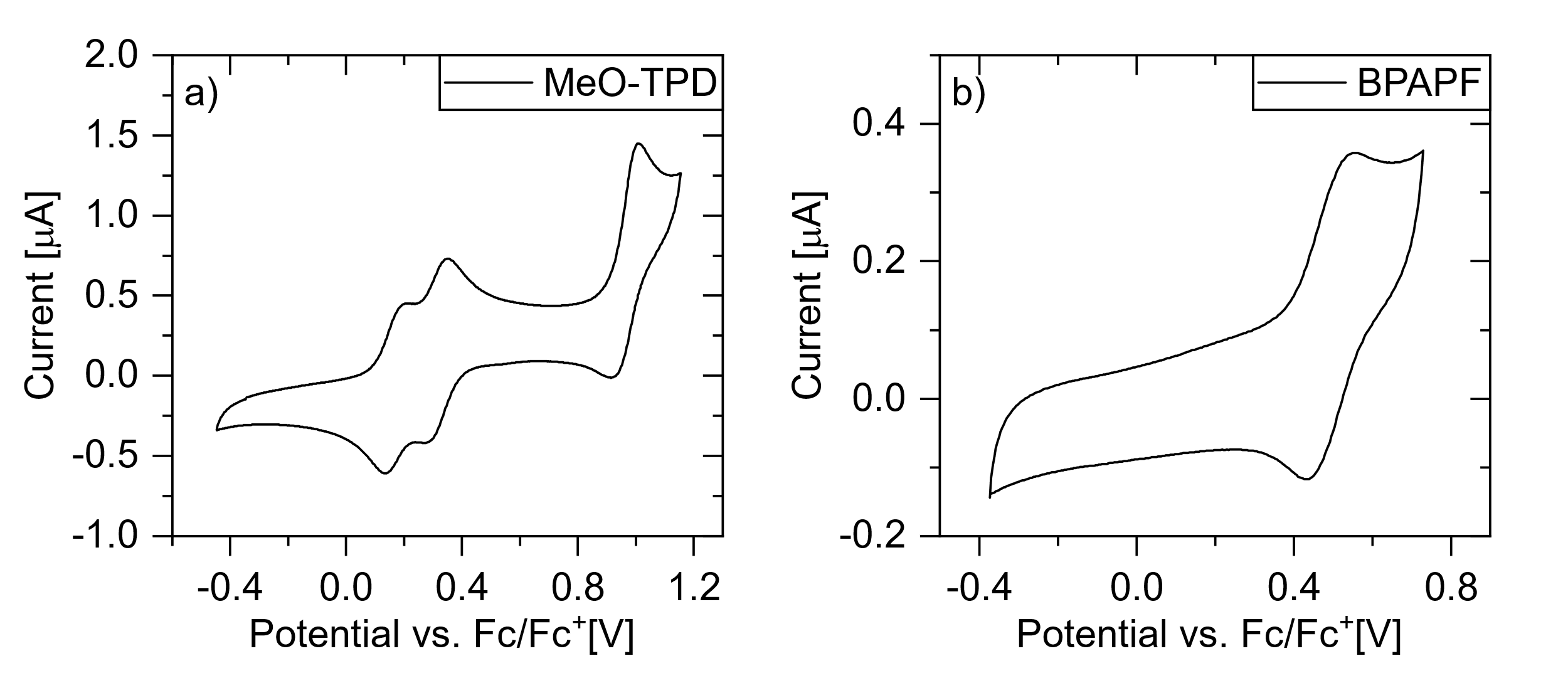


**Figure S1.** Cyclic voltammograms of a) MeO-TPD and b) BPAPF at a scan rate of 0.2 V s^-1^. For both a reversible electron transitions can be observed. In case of MeO-TPD two additional reversible transitions occur. The measurement was performed for different scan rates. By taking the average of E_1/2_ of the redox events for the different scan rates and assuming −4.8 eV as the redox potential of ferrocene, we calculate the HOMO levels of MeO-TPD (E_1/2_ =0.16 vs. Fc/Fc^+^) and BPAPF (E_1/2_ =0.49 V vs. Fc/Fc^+^) with −4.96 eV and −5.29 eV, respectively.^[14]^


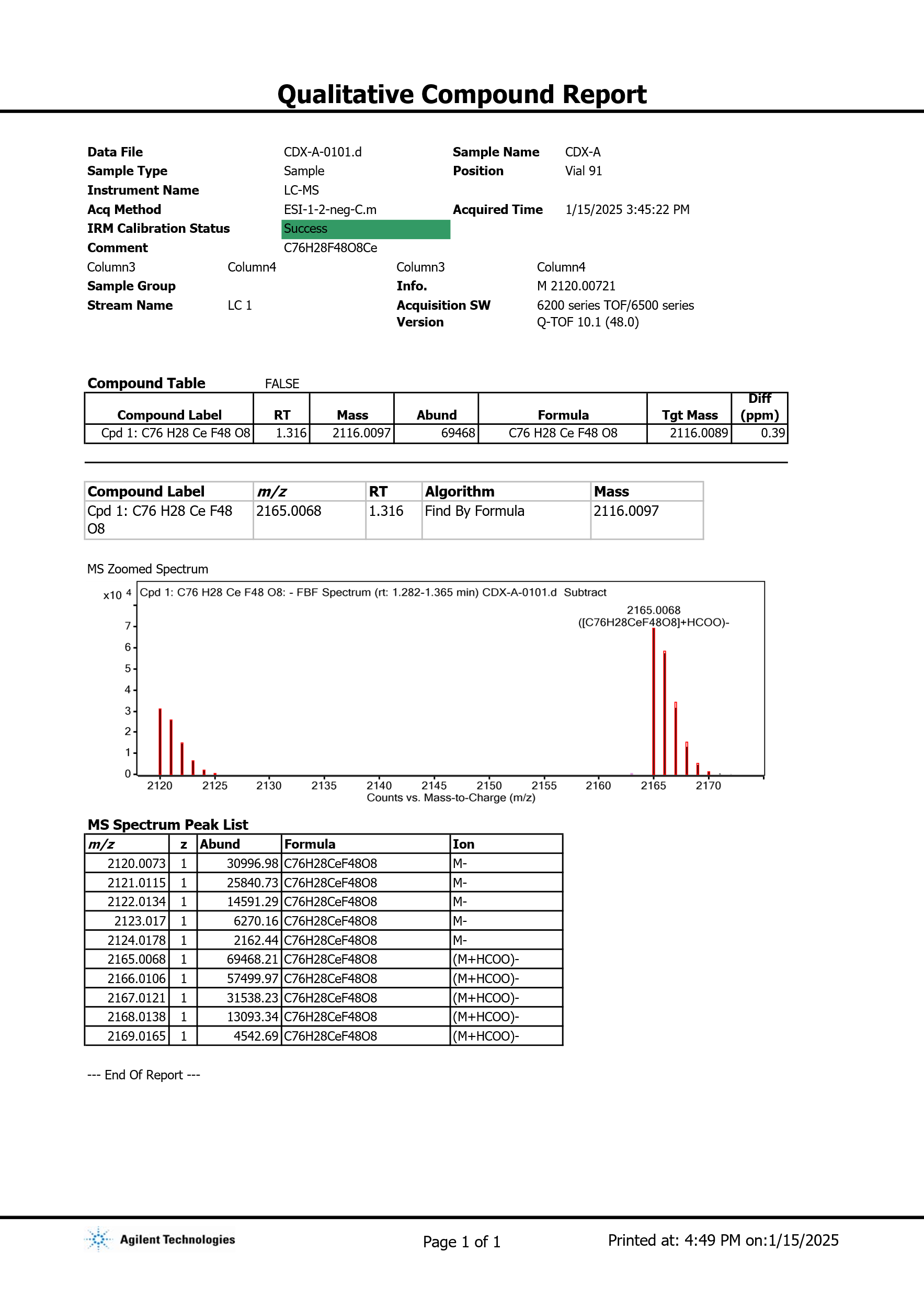


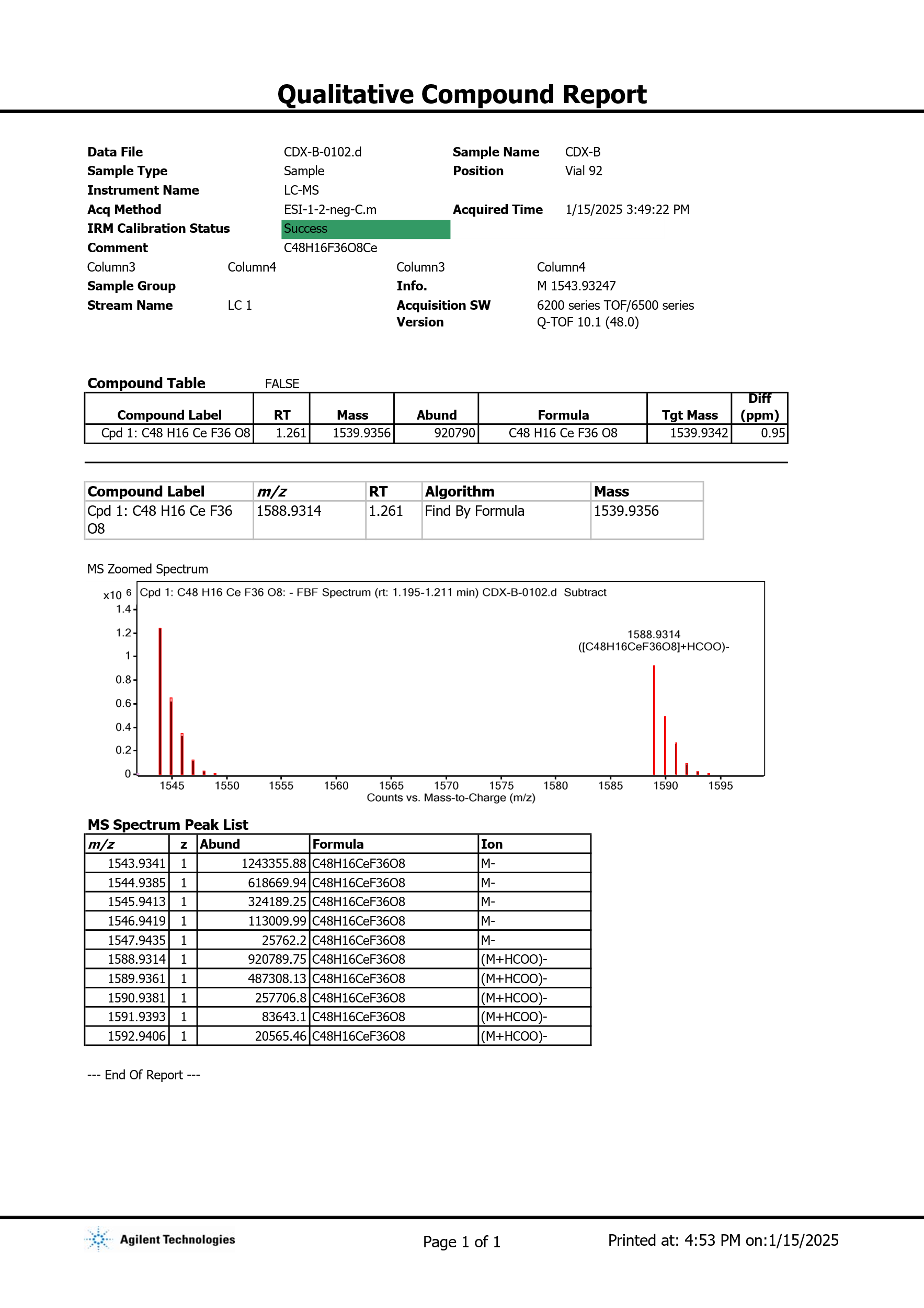

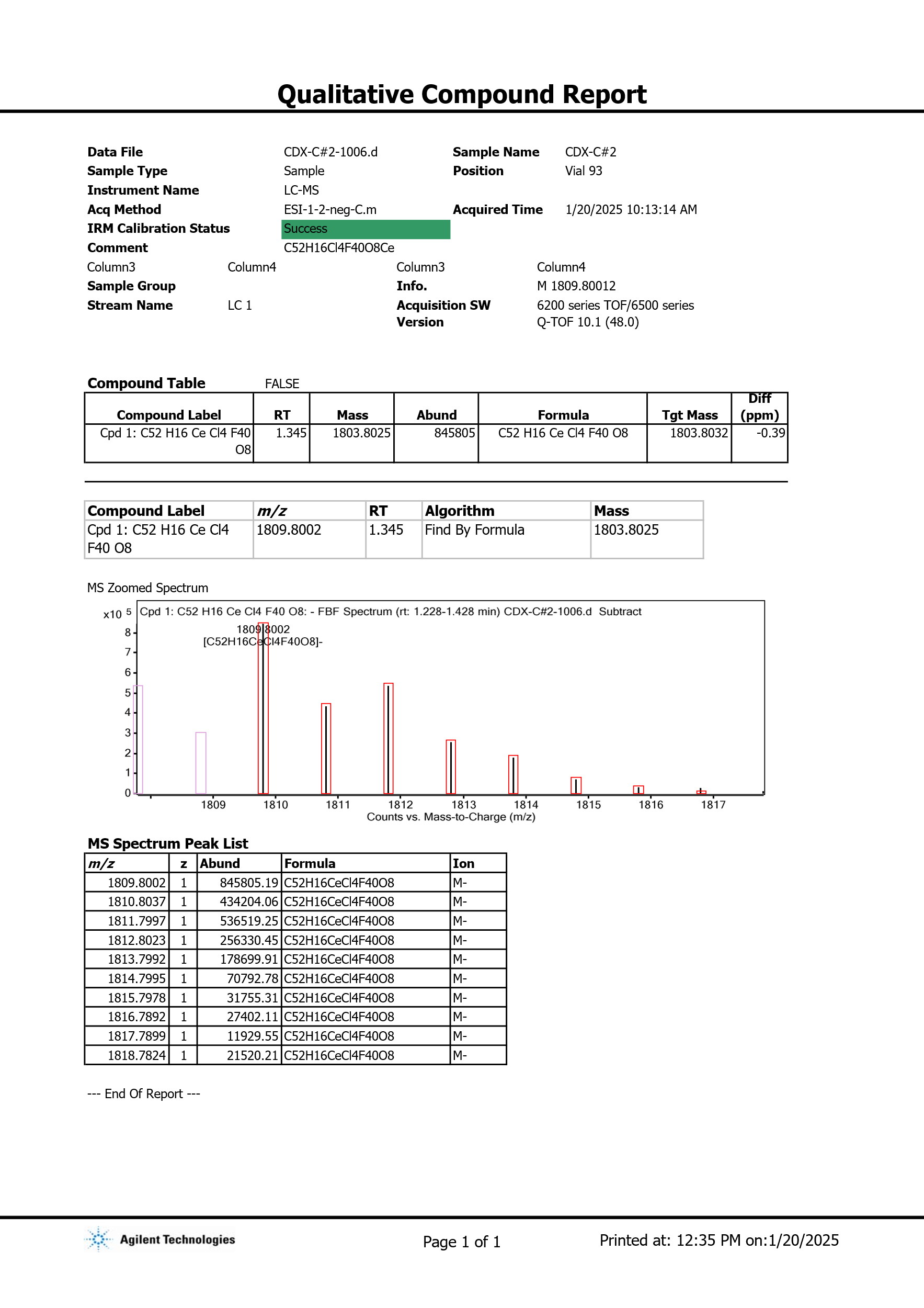


**Figure S2.** High-resolution mass spectroscopy data reports for CDX-A, CDX-B and CDX-C measured with ESI^-^ in solution with acetonitrile.


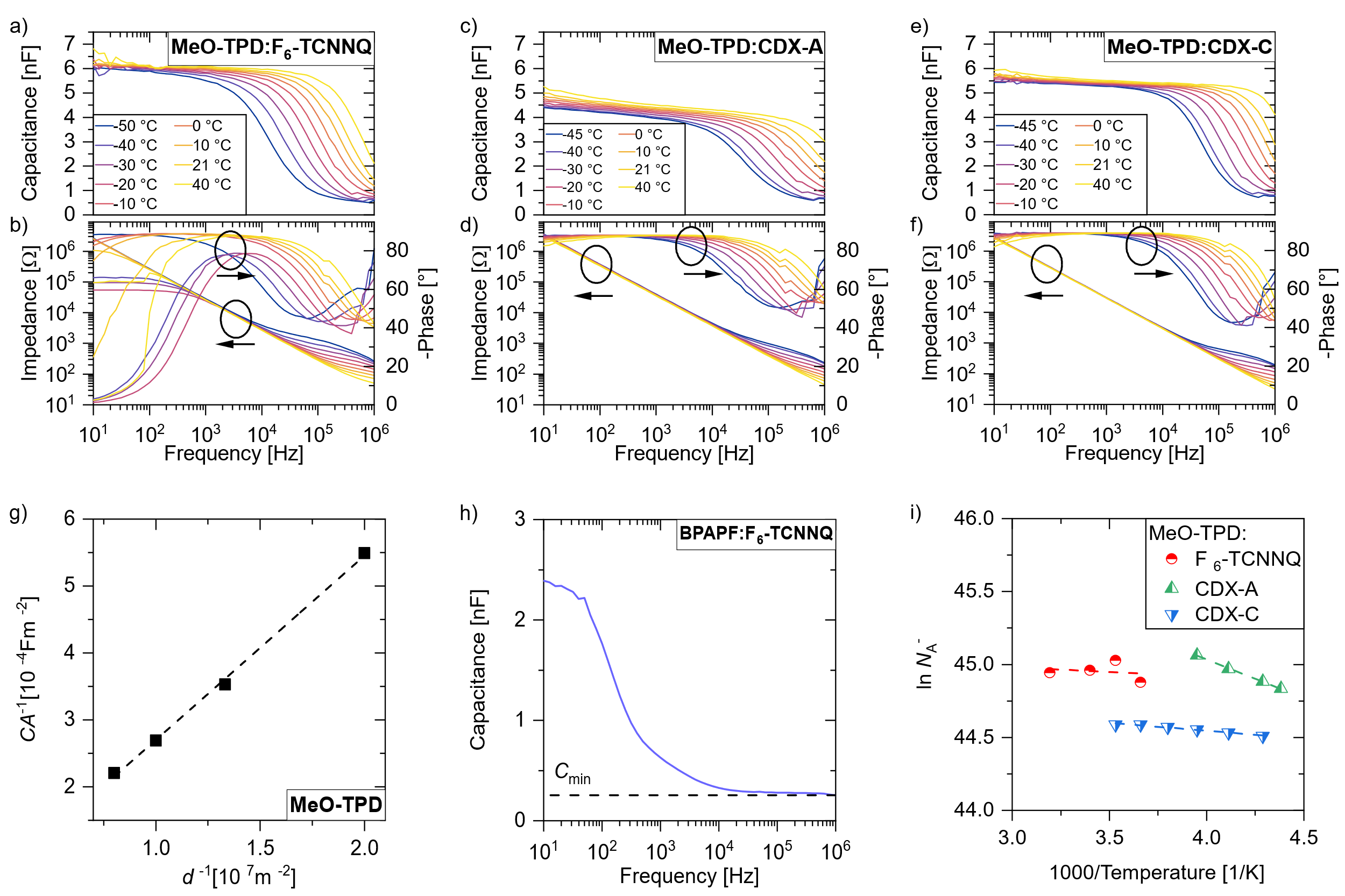


**Figure S3. a)** to **f)** Capacitance over frequency and Bode plots of thin layers of MeO-TPD doped by 1.7 mol% F_6_-TCNNQ, CDX-A, and CDX-C for various temperatures. **g)** The capacitance of neat MeO-TPD layers was measured at different layer thicknesses to calculate the dielectric function $\boldsymbol{\varepsilon}_{\boldsymbol{r}}$ by using the relation for a plate capacitor $\boldsymbol{C}\boldsymbol{=}\boldsymbol{\varepsilon}_{\boldsymbol{0}}\boldsymbol{\varepsilon}_{\boldsymbol{r}}\frac{\boldsymbol{A}}{\boldsymbol{d}}$. With a linear fit of this plot, $\boldsymbol{\varepsilon}_{\boldsymbol{r}}$ was determined with 3.1 for MeO-TPD. It is assumed, that this value is approximately the same for doped layers. **h)** Capacitance over frequency of BPAPF:F_6_-TCNNQ (15 mol%) at -30 °C. Assuming the capacity approaches the geometric capacitance of the device during the freeze-out and using the relation for a plate capacitor, we estimate a dielectric function $\boldsymbol{\varepsilon}_{\boldsymbol{r}}$ of 3.2 for this sample. The same value was used for all BPAPF samples. **i)** Logarithm of the density of ionized dopants $N_{A}^{-}$ over the inverse of temperature for MeO-TPD doped by 1.7 mol% of F_6_-TCNNQ and CDX-C. The frequency for the Mott-Schottky analysis must be the same for all evaluated temperatures, which limits the suitable temperature range for the fit due to the temperature dependency of the phase.


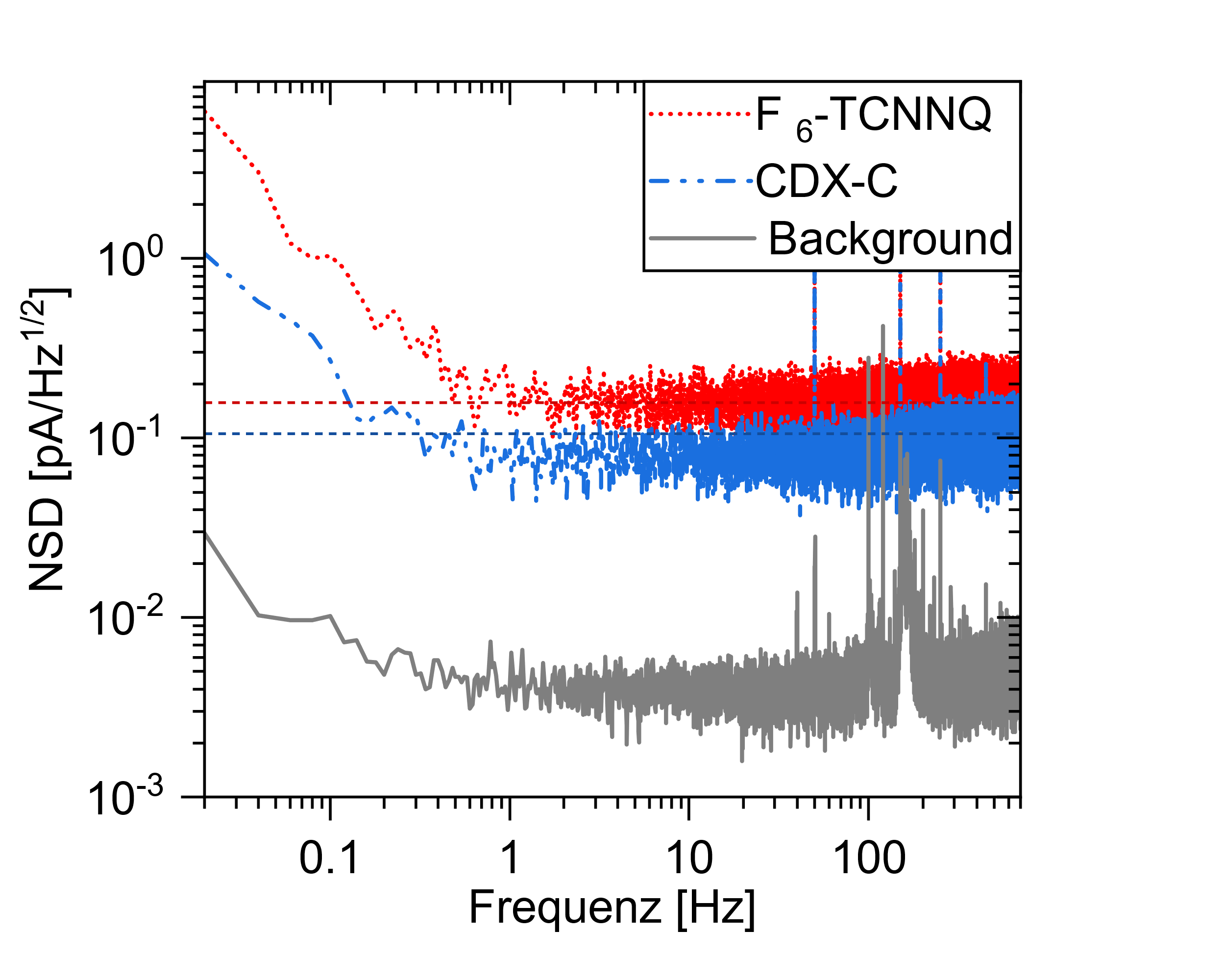


**Figure S4**. Noise spectral density (NSD) at 0 V for narrowband OPDs with the two p-dopants F_6_-TCNNQ and CDX-C in MeO-TPD as hole transport layer and background noise (grey curve). The measured noise matches the thermal noise (dashed horizontal lines), calculated from the shunt resistance of the devices obtained close to zero bias.

**
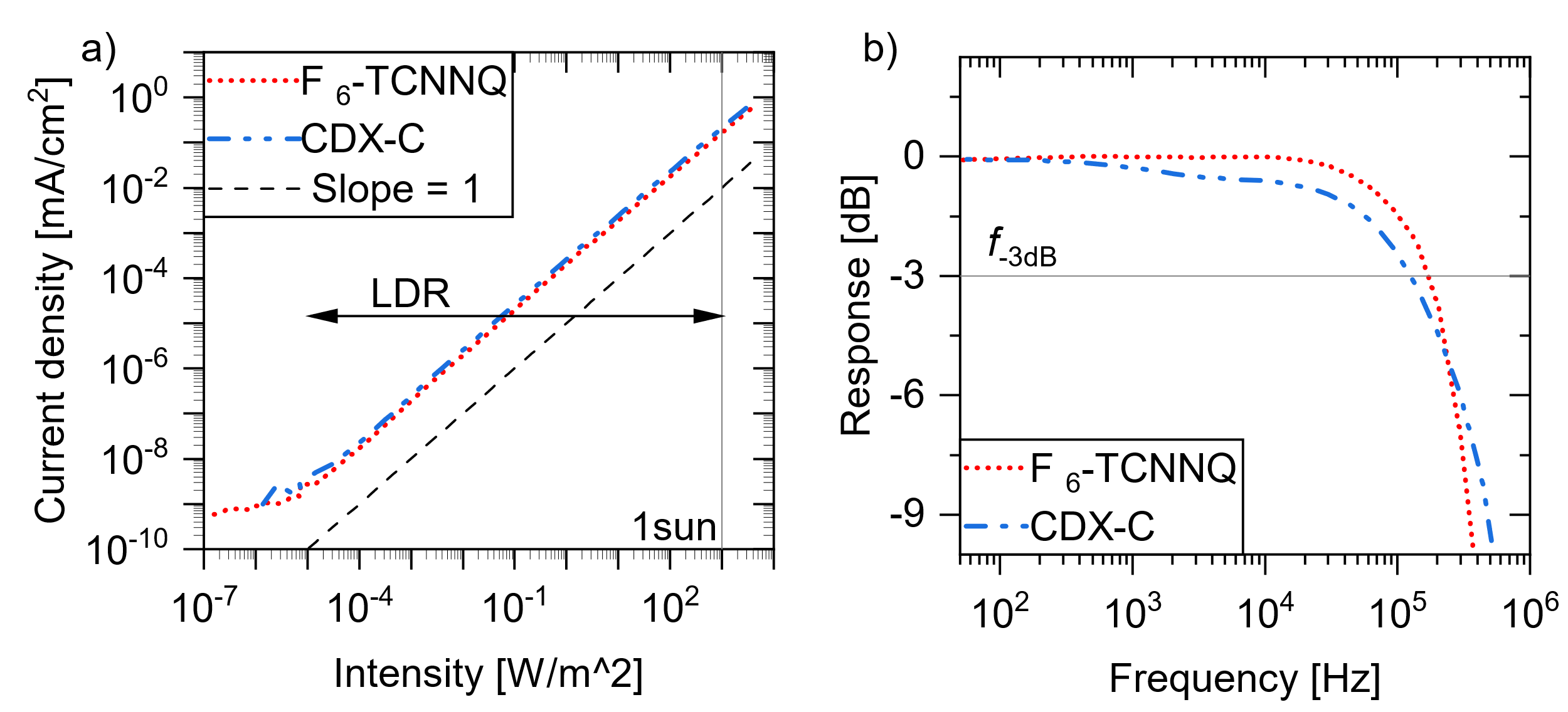
**

**Figure S5.** **a)** The linear dynamic range (LDR) of both devices is 160 dB. However, since there is only a slight deviation at intensities above 1 sun, it might be even higher if higher intensities would be measured. **b)** The transient response of both devices is similar with a cutoff frequency of 170 kHz and 125 kHz for F_6_-TCNNQ and CDX-C, respectively.
